# High Cerebral Oxygen Saturation Levels During One-Lung Ventilation Predict Better Cognitive and Clinical Outcomes After Thoracic Surgery: A Retrospective Observational Study

**DOI:** 10.3390/jpm15090445

**Published:** 2025-09-22

**Authors:** Ignacio Garutti, Francisco de la Gala, Javier Hortal, Almudena Reyes, Elena de la Fuente, David Martinez-Gascueña, Carlos Alberto Calvo, Santiago Hernández, Estrela Caamaño, Carlos Simón, Elena Vara, Patricia Piñeiro

**Affiliations:** 1 Departamento de Anestesia y Reanimación Hospital General Universitario Gregorio Marañón, Calle Doctor Esquerdo 46, 28009 Madrid, Spain; francisco.gala@salud.madrid.org (F.d.l.G.); mariaalmudena.reyes@salud.madrid.org (A.R.); efuente@salud.madrid.org (E.d.l.F.); dmgascuena@salud.madrid.org (D.M.-G.); carlosalberto.calvo@salud.madrid.org (C.A.C.); sgarciahernandez@salud.madrid.org (S.H.); patricia.pineiro@salud.madrid.org (P.P.); 2 Departamento Farmacología de la Facultad de Medicina de la Universidad Complutense Madrid Plaza de Ramón y Cajal, s/n, Ciudad Universitaria, 28040 Madrid, Spain; 3 Fundación para la Investigación del Hospital Gregorio Marañón, 28009 Madrid, Spain; carlos.simon@salud.madrid.org (C.S.); evaraami@ucm.es (E.V.); 4 Departamento Cirugía Torácica del Hospital General Universitario Gregorio Marañón, Calle Doctor Esquerdo 46, 28009 Madrid, Spain; 5 Departamento Cirugía de la Facultad de Medicina de la Universidad Complutense Madrid, Plaza de Ramón y Cajal, s/n, Ciudad Universitaria, 28040 Madrid, Spain; 6 Departamento Bioquímica de la Facultad de Medicina de la Universidad Complutense Madrid, Plaza de Ramón y Cajal, s/n, Ciudad Universitaria, 28040 Madrid, Spain

**Keywords:** one-lung ventilation, regional cerebral oxygen saturation (rScO_2_), postoperative cognitive dysfunction (POCD), thoracic surgery, permissive hypercapnia, neuroinflammation

## Abstract

**Background:** Cerebral desaturation during one-lung ventilation (OLV) in thoracic surgery has been associated with postoperative cognitive dysfunction (POCD). While the adverse effects of low intraoperative regional cerebral oxygen saturation (rScO_2_) are well documented, the potential clinical value of maintaining supranormal rScO_2_ levels has not been thoroughly studied. **Methods:** We conducted a retrospective observational study based on a previously collected cohort from a tertiary university hospital. Adult patients undergoing elective thoracic surgery between January 2019 and December 2022 were included, provided they received lidocaine either intravenously or via a paravertebral block as part of a standardized anesthetic protocol. Patients were divided into the following two groups based on their mean INVOS values 30 min into OLV: those with rScO_2_ ≥75% (H-INVOS group) and <75% (L-INVOS group). Intraoperative physiological variables, inflammatory biomarkers, cognitive function via the Mini-Mental State Examination, and postoperative outcomes were analyzed. **Results:** The H-INVOS group exhibited significantly higher preoperative lung function, higher PaO_2_ and PaCO_2_ values during OLV, and higher hemoglobin concentrations across all timepoints. They also demonstrated better preservation of cognitive function, lower IL-18 expression at 24 h postoperatively, and shorter hospital stays. There were no statistically significant differences in intraoperative hemodynamics or ventilatory mechanics.

## 1. Introduction

There is growing evidence of a relationship between regional cerebral oxygen saturation (rScO_2_) during anesthesia and postoperative cognitive dysfunction (POCD) [1]. This association is particularly relevant in thoracic surgery, where one-lung ventilation (OLV) can compromise cerebral oxygenation [2,3,4]. Consequently, intraoperative rScO_2_ monitoring has become an important strategy to reduce postoperative neurological injury.

POCD is a multifactorial and clinically significant syndrome, especially in patients over 60 years of age. Because no effective treatment is currently available, prevention is critical [3]. POCD negatively impacts patients’ quality of life, prolongs hospitalization, increases healthcare costs, and is associated with higher postoperative mortality [2].

Unlike arterial oxygen saturation measured by pulse oximetry (SpO_2_), rScO_2_ displays a wider normal range. Notably, intraoperative rScO_2_ reductions are frequent during OLV—even when SpO_2_ remains within normal limits—and have been associated with an increased risk of POCD [2,5].

The pathophysiology of POCD is thought to involve, among other mechanisms, excessive perioperative inflammation [5,6]. Cerebral hypoxia may trigger neuroinflammatory pathways and disrupt the blood–brain barrier, allowing inflammatory mediators to penetrate cerebral tissue. Similarly, systemic inflammation has been shown to promote neuroinflammation and subsequent neuronal injury [7,8]. Conversely, excessive cerebral oxygenation has also been implicated in the generation of reactive oxygen species and inflammatory mediators, which may contribute to neuronal damage [9].

In this context, intraoperative cerebral oxygenation monitoring has gained increasing attention. While cerebral desaturation is traditionally considered harmful, recent evidence suggests that abnormally elevated rScO_2_ values may also pose risks. Although the exact threshold of cerebral hyperoxia remains uncertain, studies using jugular venous oxygen saturation have proposed a cutoff of 75% [10]. Consistently, thoracic surgery studies have suI´ve doneggested that cerebral hyperoxia may occur when rScO_2_ exceeds this threshold [11]. Based on this evidence, we used 75% as a pragmatic cutoff to investigate the potential association between intraoperative cerebral hyperoxia and adverse neurological outcomes. From a personalized medicine perspective, monitoring rScO_2_ provides real-time, patient-specific information that can guide individualized anesthetic management, moving beyond population-based thresholds and toward tailored perioperative care.

The aim of this study was to evaluate the postoperative impact of maintaining elevated In Vivo Optical Spectroscopy (INVOS) values compared with lower values, with a primary focus on prognostic variables such as length of hospital stay.

## 2. Materials and Methods

### 2.1. Study Design

This study is a secondary analysis of data collected within the framework of the original trial (NCT03905837, EudraCT 2016-004271-52), a prospective study conducted under a predefined protocol and approved by the Ethics Committee of Gregorio Marañón Hospital, Madrid, Spain (Chairperson: Dr. Camino Sorabe; protocol code IGMFGG-2016; approval date: 3 April 2018). The original study was conducted in accordance with the Declaration of Helsinki.

The objective of this subanalysis is to evaluate the relationship between intraoperative cerebral oxygenation levels and postoperative clinical, inflammatory, and neurocognitive outcomes.

### 2.2. Study Population

Adult patients scheduled for thoracic surgery between January 2019 and December 2022 were eligible. The original trial enrolled 154 patients randomized into the following three groups: (1) paravertebral lidocaine (2 mg·kg^−1^·h^−1^) plus intravenous saline, (2) intravenous lidocaine (1.5 mg·kg^−1^·h^−1^) plus paravertebral saline, and (3) intravenous remifentanil (0.1 µg·kg^−1^·h^−1^) plus paravertebral saline. The primary endpoint of the parent study was the incidence of postoperative complications according to the Clavien–Dindo classification.

For the present analysis, only patients receiving lidocaine (intravenous infusion or paravertebral block) as part of the standardized anesthetic protocol were included. The control group without lidocaine was excluded to avoid confounding effects on inflammatory profiles. The exclusion criteria were pre-existing cognitive impairment, active neurological disease, emergency thoracic surgery, missing intraoperative INVOS monitoring data, and absence of postoperative cognitive assessments.

### 2.3. Anesthetic Protocol and Monitoring

No premedication was administered prior to induction. Anesthesia was induced with propofol (2–3 mg/kg), fentanyl (3–5 µg/kg), and rocuronium (1.2 mg/kg). Airway management employed a left-sided double-lumen tube or, in selected cases, a bronchial blocker; placement was confirmed with fiberoptic bronchoscopy. A radial artery catheter was inserted contralateral to the surgical side, and patients were positioned in the lateral decubitus position. A paravertebral catheter was placed at the T5–T6 level.

### 2.4. Ventilation Strategy

During two-lung ventilation, initial settings included FiO_2_ 0.4, a respiratory rate of 12 breaths/min, a tidal volume (TV) of 8 mL/kg, and optimal PEEP determined via an alveolar recruitment maneuver. With the initiation of one-lung ventilation (OLV), TV was reduced to 4–6 mL/kg, followed by a second recruitment maneuver to reassess the optimal PEEP. FiO_2_ was titrated to maintain SpO_2_ > 90%. In cases of hypoxemia (SpO_2_ < 90% with FiO_2_ > 0.8), lung isolation device placement was rechecked and continuous positive airway pressure (CPAP) was applied to the dependent lung. If a bronchial blocker was used, reinflation of the collapsed lung was attempted.

### 2.5. Patient Grouping

Patients were categorized into the following two groups according to their mean INVOS values measured 30 min after OLV initiation: ≥75% (High INVOS group) and <75% (Low INVOS group).

### 2.6. Assessment of rScO_2_ and Cognitive Function

Preoperative regional cerebral oxygen saturation (rScO_2_) was measured immediately prior to induction using bihemispheric INVOS 5100 optodes (Covidien Germany GmbH, Neustadt, Germany). Sensors were applied to the forehead bilaterally and covered with opaque plastic to minimize light interference. The mean of both hemispheres was used for analysis. Anesthetic management was not guided by absolute rScO_2_ values.

Cognitive function was assessed with the Mini-Mental State Examination (MMSE), administered preoperatively on the ward and repeated 72 h postoperatively. Both absolute values and percentage changes from baseline were analyzed.

### 2.7. Data Collection

Measurements were obtained at the following three intraoperative timepoints:

1. After lateral positioning, prior to OLV initiation.
2. Thirty minutes after OLV initiation, once respiratory parameters had stabilized.
3. Fifteen minutes after resuming two-lung ventilation.

At each timepoint, arterial blood gases, bronchoalveolar lavage samples, and blood samples for inflammatory biomarkers were collected. Airway pressures and pulmonary compliance were recorded. Hemodynamic variables were obtained via pulse contour analysis (FloTrac), including blood pressure, stroke volume, cardiac output, and stroke volume variation.

The inflammatory biomarkers assessed included TNF-α, IL-1, IL-6, IL-18, IL-10, MMP-2, MMP-3, and MMP-9. Markers of neurological injury were also measured. Additional blood samples for the same analyses were drawn 24 h postoperatively.

### 2.8. Postoperative Course

Patients were transferred to the post-anesthesia care unit and discharged after 24 h if hemodynamic and respiratory parameters were stable. Postoperative complications were assessed at 30 days and classified according to the Clavien–Dindo system [12]. Evaluators were blinded to intraoperative clinical data.

### 2.9. Statistical Analysis

Data were analyzed with SPSS version 25.0. Continuous variables are reported as medians with interquartile ranges (IQR 25–75), and categorical variables as absolute frequencies and percentages. Between-group comparisons were performed using the Mann–Whitney U test for continuous variables and Chi-square or Fisher’s exact tests for categorical variables. Repeated-measures analysis was applied to evaluate changes in continuous variables over time. Kaplan–Meier analysis was used to compare outcomes between the High and Low INVOS groups. A *p*-value of <0.05 was considered statistically significant.

## 3. Results

### 3.1. Baseline Characteristics

A total of 98 patients were included in the analysis. The median age was 67 years (IQR 62–72), and 64% were male. Patients with elevated INVOS values during OLV showed better preoperative pulmonary function test results; however, no significant differences were observed between groups with respect to baseline comorbidities (Table 1).

Arterial blood gas analysis during OLV revealed higher PaO_2_ values and PaO_2_/FiO_2_ ratios in patients in the H-INVOS group. Hemoglobin concentrations were also consistently higher at all three assessed time points. No significant differences were observed between groups in terms of mechanical ventilation settings or hemodynamic parameters (Table 2).

**Figure 1:**
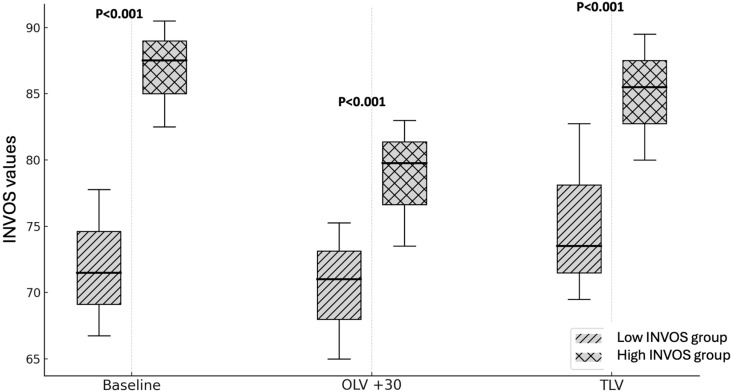
INVOS values during surgery (high vs. low INVOS groups). OLV + 30: After 30 min of one-lung ventilation; TLV: Two-lung ventilation; *p* < 0.001 High INVOS vs. Low INVOS groups.

Patients in the H-INVOS group exhibited consistently higher rScO_2_ values not only during OLV, but also before and after the OLV phase (Figure 1).

### 3.2. Inflammatory Biomarkers

**Figure 2:**
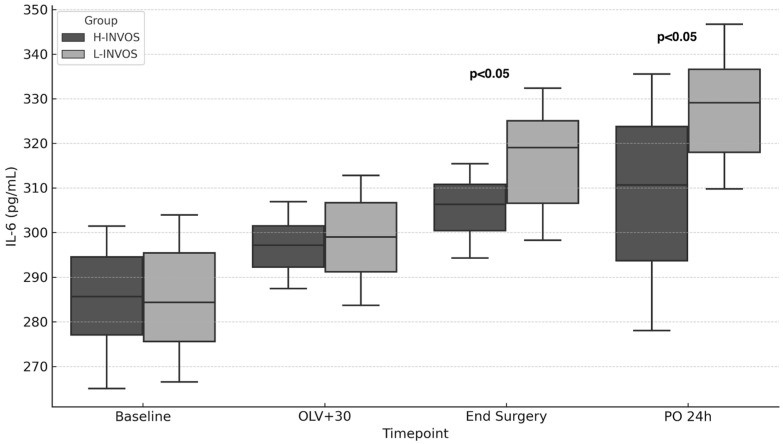
IL-6 levels over time by INVOS group. Footnote: OLV + 30: After 30 min of one-lung ventilation; TLV: Two-lung ventilation; PO: Postoperative. *p* < 0.05: High INVOS vs. Low INVOS groups.

Most inflammatory biomarkers were comparable between groups, with the exception of IL-6. Patients in the L-INVOS group had significantly higher IL-6 levels both at the end of surgery and on postoperative day 1 compared with the H-INVOS group (Figure 2, Table 3).

### 3.3. Cognitive Outcomes and Length of Hospital Stay

Preoperative MMSE scores were comparable between groups. At 72 h postoperatively, however, MMSE scores were significantly lower in the L-INVOS group compared with the H-INVOS group (30 [IQR 29–30] vs. 29 [28–30], *p* = 0.018). The percentage decline in MMSE scores was likewise greater in the L-INVOS group (Table 4).

**Figure 3:**
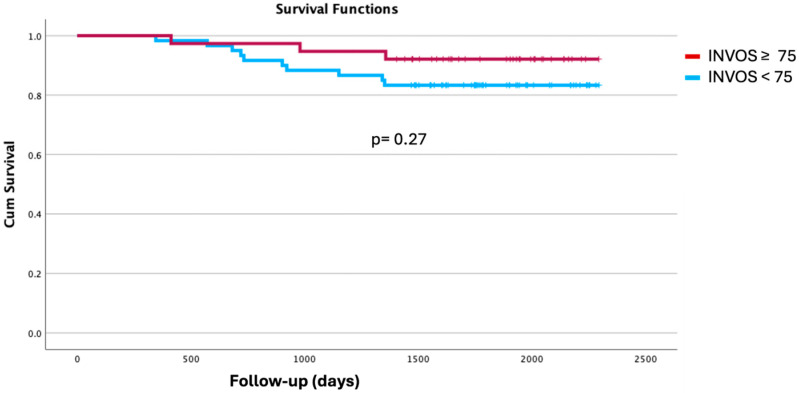
Kaplan Meier curves. INVOS ≥ 75 vs. <75 during one-lung ventilation.

Length of hospital stay was significantly shorter in the H-INVOS group (median 4 [IQR 3–6] days) compared with the L-INVOS group (5 [4–6.5] days; *p* = 0.019). Although no statistically significant differences were observed in the distribution of Clavien–Dindo complication grades, 71% of patients in the H-INVOS group experienced no complications versus 55% in the L-INVOS group (*p* = 0.112). All four cases of major postoperative complications occurred in the L-INVOS group. No significant differences were found in the detailed analysis of postoperative complications (Table 4). Kaplan–Meier survival analysis likewise showed no significant differences between groups (log-rank *p* = 0.27; Figure 3).

## 4. Discussion

Our findings suggest that maintaining intraoperative INVOS values ≥ 75% during OLV with a lung-protective ventilation strategy is associated with improved neurocognitive outcomes, shorter hospital stays, and attenuation of the inflammatory response—particularly reflected by lower IL-6 levels.

### 4.1. Cerebral Oxygenation and Postoperative Cognitive Function

These results support the hypothesis that adequate cerebral oxygenation during OLV plays a critical role in preserving postoperative cognitive function. Previous studies have shown that rScO_2_ often declines during OLV—primarily due to reductions in PaO_2_—in 30% to 100% of patients [4,13,14,15]. Such decreases have been consistently associated with an increased risk of POCD [2,11,16]. Consequently, thoracic anesthesia practice has progressively emphasized not only maintaining adequate peripheral oxygen saturation (SpO_2_), but also avoiding intraoperative cerebral desaturation, particularly during OLV.

Most published guidelines recommend maintaining rScO_2_ above 65% during OLV to reduce the risk of POCD. In our study, we investigated the impact of targeting an even higher threshold. This decision was based on the physiological rationale that permissive hypercapnia—a key component of lung-protective ventilation—may enhance cerebral blood flow and thereby increase cerebral tissue oxygenation [17]. We considered the ≥75% threshold both safe for preventing cerebral hypoxia and sufficiently conservative to avoid cerebral hyperemia [11].

Consistent with previous reports, our data showed that patients with higher intraoperative rScO_2_ values during OLV demonstrated a better postoperative cognitive performance on MMSE assessments, with fewer patients exhibiting scores below 26 points or declines greater than 2 points. Although these differences reached statistical significance, they should be interpreted with caution, as the MMSE—despite its widespread clinical use—remains a relatively crude and nonspecific tool for detecting subtle postoperative cognitive changes. It is characterized by a high specificity but limited sensitivity for identifying POCD.

### 4.2. Permissive Hypercapnia

Evidence on the impact of rScO_2_ levels under lung-protective ventilation strategies incorporating low tidal volumes and permissive hypercapnia remains limited. PaCO_2_ is a potent regulator of cerebral blood flow, inducing cerebral vasodilation, increasing intracranial blood volume, and consequently elevating rScO_2_. Végh et al. reported that, in normocapnic patients ventilated with a constant PEEP of 5 cmH_2_O, cerebral desaturation during OLV was uncommon, although 20% of patients still experienced decreases in rScO_2_ below 60% [18]. By contrast, in our cohort—managed under clear hypercapnic conditions—only 3% of patients had rScO_2_ values below 60% during OLV. Moreover, the mean reduction in rScO_2_ observed in our study was comparable to that reported by Végh and colleagues. Therefore, we suggest that their conclusion—that maintaining normocapnia improves cerebral oxygenation—may not be generalizable. Instead, the lower incidence of severe desaturation in our population may largely reflect the use of permissive hypercapnia.

In addition, unlike other studies, we did not find a consistent association between cerebral desaturation and variables such as ventilatory pressures [3,19] and hemodynamic instability [16]. These discrepancies may be explained by differences in study design, particularly the use of lung-protective ventilation with resultant permissive hypercapnia, although the influence of other uncontrolled factors cannot be ruled out.

### 4.3. Cerebral Oxygen Threshold

In thoracic anesthesia practice, the effective interpretation of cerebral oximetry requires clear and standardized thresholds that can be applied consistently across patients. One of the main limitations of rScO_2_ monitoring is the absence of a simple, universally accepted cutoff values for clinically significant desaturation—values that would prompt corrective interventions during OLV, such as increasing FiO_2_, applying CPAP to the non-dependent lung, or performing recruitment maneuvers. However, indiscriminate application of these strategies carries risks, including oxidative stress, surgical field interference, and barotrauma.

This challenge is compounded by wide interindividual variability in rScO_2_ values, influenced by factors such as age, cerebral anatomy, cranial bone thickness, systemic perfusion, and head positioning. Consequently, many authors advocate for using relative rScO_2_ changes during OLV—commonly defined as reductions ≥15–20% from baseline—as markers of clinically relevant desaturation [14,16,20,21]. Others support absolute thresholds [2,13] or recommend a combined approach that incorporates both absolute and relative criteria [22]. From a practical standpoint, we consider that relying solely on relative changes may complicate bedside decision making and fails to provide a straightforward clinical signal to guide intervention.

### 4.4. SpO_2_ vs. rScO_2_

In our study, SpO_2_ values did not differ significantly between groups. Nevertheless, patients in the H-INVOS group demonstrated superior arterial oxygenation and gas exchange, as evidenced by higher PaO_2_/FiO_2_ ratios. These findings reinforce the concept that pulse oximetry alone is not a reliable surrogate for monitoring cerebral oxygenation.

### 4.5. Neuroinflammation and Its Role in Postoperative Cognitive Dysfunction

POCD is multifactorial in origin, with perioperative inflammation recognized as a major pathogenic contributor. Thoracic surgery elicits a substantial systemic inflammatory response, driven both by surgical trauma and by OLV-induced lung injury [23,24]. It is well established that the perioperative release of proinflammatory mediators compromises blood–brain barrier integrity, triggering endothelial dysfunction and the infiltration of peripheral immune cells into the brain parenchyma. These processes activate astrocytes and microglia, leading to neuronal dysfunction, memory impairment, and ultimately POCD [25,26]. Interleukin-6 (IL-6) has been identified as a key cytokine in postoperative brain injury, modulating neuronal plasticity and synaptic activity in the hippocampus and cerebral cortex—regions critical for executive function [27].

Wang et al. demonstrated that the correction of cerebral desaturation events was associated with reduced perioperative inflammatory markers and lower rates of POCD [28]. Additional studies have shown that peripheral inflammation can lead to neuroinflammation and subsequent cerebral tissue damage [29,30].

In our study, preoperative inflammatory biomarkers did not differ significantly between groups. However, patients with lower intraoperative rScO_2_ values during OLV exhibited higher IL-6 levels at the end of surgery and again 24 h later [25,31,32]. Conversely, reduced IL-6 expression in patients with higher cerebral oxygenation suggests that a more stable perfusion and oxygenation environment may attenuate this neuroinflammatory cascade.

It should be noted, however, that this subanalysis included only patients who received perioperative lidocaine, a drug with well-documented anti-inflammatory properties. Therefore, we cannot exclude the possibility that lidocaine contributed to the absence of significant differences in other neuroinflammatory markers.

### 4.6. Cerebral Oximetry as a Systemic Indicator and Predictor of Recovery

The extracerebral significance of regional cerebral oxygen saturation (rScO_2_) monitoring during thoracic surgery remains incompletely understood. In our study, patients with lower intraoperative rScO_2_ values during OLV experienced a longer hospital stay. This finding has important clinical implications, as prolonged hospitalization increases the risk of nosocomial complications and is unequivocally associated with higher healthcare costs.

Although rScO_2_ monitoring was initially developed to optimize cerebral outcomes, the brain may serve as a sentinel or “index organ”, reflecting global tissue oxygenation. Given the brain’s robust autoregulatory capacity, it may be the last organ to manifest signs of compromised perfusion or oxygen delivery. This suggests that rScO_2_ thresholds currently considered acceptable may, in fact, need to be set at supranormal levels to adequately reflect systemic oxygenation. Accordingly, significant cerebral desaturations may imply concurrent desaturation in other organs, potentially contributing to the broad range of adverse perioperative outcomes described in prior studies [14,21].

The mechanism linking higher cerebral oxygenation with improved clinical recovery may involve preserved cognitive function, which facilitates early mobilization, adherence to postoperative treatment, and overall functional rehabilitation. Although the overall incidence of postoperative complications was similar between groups, there was a trend toward fewer pulmonary complications and a lower frequency of Clavien–Dindo grade ≥2 events in patients with higher INVOS values during OLV.

These findings are consistent with those of Kazan et al., who associated persistently low cerebral oxygenation with an increased risk of respiratory complications [14]. In line with Roberts’ observations, we propose that rScO_2_ may function as a marker of global recovery potential, capturing perioperative vulnerability not adequately reflected in conventional classifications such as Clavien–Dindo. In this sense, rScO_2_ may be considered not only as a neurological monitoring parameter, but also as a biomarker for personalized perioperative medicine, helping to stratify risk, predict recovery trajectories, and guide individualized interventions.

## 5. Limitations

This study has several limitations. First, it was a retrospective subanalysis, with the inherent methodological constraints of such a design. Although strict inclusion criteria were applied and the non-lidocaine group was excluded to minimize pharmacological confounding, residual confounding cannot be entirely ruled out. Second, while the sample size was sufficient to detect differences in primary outcomes such as MMSE scores, it may have been underpowered to identify differences in less frequent outcomes, including major complications and mortality. Third, although the MMSE is widely used, it has a limited sensitivity for detecting subtle postoperative cognitive changes. Moreover, we did not include systematic monitoring for potential complications associated with elevated rScO_2_ values, nor did we employ a comprehensive neuropsychological battery that would have allowed for a more detailed assessment of the specific cognitive domains affected.

## 6. Conclusions

Although the optimal absolute rScO_2_ threshold remains a matter of debate, our findings suggest that higher intraoperative values may help identify patients at lower risk of complications and support faster recovery. Beyond its neurological relevance, rScO_2_ may also serve as a surrogate marker of global physiological status, particularly in the setting of lung-protective strategies such as permissive hypercapnia. Prospective studies are warranted to confirm these observations and to determine whether rScO_2_-guided interventions can improve perioperative outcomes. Our results suggest that incorporating cerebral oximetry into routine perioperative care could contribute to a more personalized approach, in which anesthetic and ventilatory strategies are adapted to the patient’s unique physiological response, ultimately improving outcomes.

## Figures and Tables

**Table 1:** Baseline characteristics of patients according to INVOS values.

Variable		INVOS ≥ 75 (*n* = 38)	INVOS ≤ 75 (*n* = 60)	*p*-Value
Age (years)		62 [55.5–71.5]	64 [60–75]	0.185
YesWeight (kg)		80 [67–84]	71 [61–85]	0.190
Height (cm)		165 [160–175]	165 [160–170.5]	0.385
Ideal Body Weight (kg)		60 [55–71.5]	60 [54.5–66]	0.453
BMI (kg/m^2^)		27.11 [23.73–31.2]	26.94 [23.52–31.45]	0.766
ASA Class	I	2 (5.3%)	1 (1.7%)	0.439
	II	17 (44.7%)	21 (35%)	
	III	18 (47.4%)	37 (61.7%)	
	IV	1 (2.6%)	1 (1.7%)	
FEV1 (% predicted)		100.5 [86–114.75]	87 [72–98]	0.002
FVC (% predicted)		105 [99–115]	96 [89–106]	0.017
FEV_1_/FVC Ratio		0.75 [0.68–0.82]	0.7 [0.55–0.76]	0.028
DLCO (% predicted)		87 [77–108]	87.5 [76.7–104.2]	0.925
Male sex		27 (71.1%)	32 (53.3%)	0.126
ARISCAT Score		50 [40–50]	50 [43–50]	0.296
Dyslipidemia		14 (36.8%)	19 (31.7%)	0.486
Type 2 Diabetes		6 (15.8%)	9 (15%)	0.639
Type 1 Diabetes		1 (2.6%)	0 (0%)	0.168
Cardiovascular Disease		9 (23.7%)	22 (36.7%)	0.209
Arterial Hypertension		16 (42.1%)	32 (53.3%)	0.782
Respiratory Disease		9 (23.7%)	22 (36.7%)	0.087
COPD		7 (18.4%)	15 (25%)	0.181
OSA		3 (7.9%)	3 (5%)	0.344
Asthma		0 (0%)	5 (8.3%)	0.112
Renal Disease		2 (5.3%)	2 (3.3%)	0.432
Liver Disease		2 (5.3%)	3 (5%)	0.570
Psychiatric Disorder		1 (2.6%)	6 (10%)	0.229
Right side surgery		22 (57.9%)	40 (66.6%)	0.524
Type of surgery				0.737
Wedge resection		19 (50%)	23 (38.3%)	
Lobectomy		19 (50%)	36 (60%)	
Bilobectomy		0 (0%)	1 (1.7%)	
Length OLV (min)		170 [106–195]	175 [104–201]	0.789
Crystalloids (mL)		547 [472–772]	600 [500–733]	0.945

Abbreviations: INVOS = Intraoperative cerebral oximetry index (Near-Infrared Spectroscopy); BMI = Body Mass Index; ASA = American Society of Anesthesiologists physical status classification; FEV_1_ = Forced Expiratory Volume in one second; FVC = Forced Vital Capacity; DLCO = Diffusing capacity of the lung for carbon monoxide; ARISCAT = Assess Respiratory Risk in Surgical Patients in Catalonia; COPD = Chronic Obstructive Pulmonary Disease; OSA = Obstructive Sleep Apnea.

**Table 2:** Intraoperative physiologic parameters according to INVOS values at different time points.

Variable		BASELINE	*p*-Value	OLV + 30	*p*-Value	END SURGERY	*p*-Value
PaO_2_ (mmHg)	INVOS ≥ 75	184 [162–216]	0.622	97 [77–125]	0.037	189.5 [161.2–210.8]	0.170
	INVOS < 75	169 [133.5–225]		83 [71.8–100.2]		199.5 [171–249]	
FiO_2_	INVOS ≥ 75	0.5 [0.5–0.6]	0.223	0.7 [0.6–0.8]	0.080	0.6 [0.5–0.6]	0.123
	INVOS < 75	0.6 [0.5–0.6]		0.8 [0.7–0.9]		0.6 [0.6–0.7]	
PAFI	INVOS ≥ 75	341 [306–396]	0.199	124 [103–181]	0.036	333 [281–374]	0.743
	INVOS < 75	321 [257–382]		112 [86–144]		318 [254–391]	
SaO_2_ (%)	INVOS ≥ 75	100 [99–100]	0.659	96 [93–98]	0.060	100 [99–100]	0.542
	INVOS < 75	99 [99–100]		94 [91–97]		100 [99–100]	
SpO_2_ (%)	INVOS ≥ 75	98 [97–99]	0.896	95 [93–96]	0.123	98 [97–99]	0.074
	INVOS < 75	98 [97–99]		93.5 [91.8–95]		99 [98–100]	
PaCO_2_ (mmHg)	INVOS ≥ 75	52 [47–55]	0.058	57 [52–64]	0.303	54.5 [50.8–60]	0.175
	INVOS < 75	48 [44–54]		55 [50–61]		53 [47.8–61]	
Hb (g/dL)	INVOS ≥ 75	14 [12.7–14.9]	0.004	14 [13.2–15.2]	0.004	13.85 [12.8–15.3]	0.021
	INVOS < 75	12.7 [11.83–13.8]		13 [11.4–13.87]		13.1 [11.5–14.1]	
MAP (mmHg)	INVOS ≥ 75	71.5 [66–81.25]	0.439	79 [70–86.5]	0.663	75 [67–85]	0.806
	INVOS < 75	75 [64–84]		78 [68.50–87]		74 [68–84]	
HR (b/min)	INVOS ≥ 75	67 [61–73.25]	0.269	72 [64.50–80]	0.237	73 [66–79]	0.504
	INVOS < 75	65 [58.5–73.5]		68 [60.5–73]		68 [63–76.5]	
CI (L min m^2^)	INVOS ≥ 75	2.67 [2.24–3.1]	0.075	3.2 [2.51–3.38]	0.221	3.9 [2.57–3.46]	0.658
	INVOS < 75	2.5 [22–2.91]		2.77 [2.21–38]		3.1 [2.38–3.59]	
SVV (%)	INVOS ≥ 75	10 [7–13]	0.060	6.5 [4–11]	0.324	9 [7–11.5]	0.262
	INVOS < 75	12 [8–16]		6 [5–11]		9 [7–13]	
Ppeak (CmH_2_O)	INVOS ≥ 75	19 [18–21]	0.580	22 [20–26]	0.365	21 [19–24]	0.748
	INVOS < 75	19.5 [18–21]		22.5 [20–26]		22 [19–25]	
Pplateau (CmH_2_O)	INVOS ≥ 75	15 [15–17]	0.105	17 [16–21]	0.966	18 [15–19]	0.571
	INVOS < 75	16 [15–17.75]		18 [16–20]		18 [17–20]	
PEEP (CmH_2_O)	INVOS ≥ 75	5 [5]	0.773	8 [6.25–8]	0.660	8 [6–8]	0.410
	INVOS < 75	5 [5]		8 [6–8]		8 [7,8]	
TV (mL)	INVOS ≥ 75	475 [437–545]	0.926	382 [350–420]	0.499	480 [450–540]	0.588
	INVOS < 75	480 [430–532]		380 [350–425]		475 [430–525]	
Respiratory rate (/min)	INVOS ≥ 75	12 [12–13]	0.430	13 [12–14]	0.157	13 [12–14]	0.539
	INVOS < 75	12 [12–13]		13 [12–14]		13 [12–14]	

Abbreviations: INVOS = Intraoperative cerebral oximetry index; OLV = One-lung ventilation; PaO_2_ = Partial pressure of arterial oxygen; FiO_2_ = Fraction of inspired oxygen; PAFI = PaO_2_/FiO_2_ ratio; SaO_2_ = Arterial oxygen saturation; SpO_2_ = Peripheral oxygen saturation; PaCO_2_ = Partial pressure of arterial carbon dioxide; Hb = Hemoglobin; MAP = Mean arterial pressure; HR = Heart rate; CI = Cardiac index; SVV = Stroke volume variation; Ppeak = Peak airway pressure; Pplateau = Plateau pressure; PEEP = Positive end-expiratory pressure; TV = Tidal volume.

**Table 3:** Biomarker levels at different time points according to INVOS values.

**Variable**		**Baseline**	***p***	**OLV + 30**	***p***	**End Surgery**	***p***	**24 h PO**	***p***
NSE (IU)	INVOS ≥ 75	14 [13–15]	0.57	14.3 [13–15]	0.800	14.9 [13.8–15.5]	0.836	14.2 [13.5–14.9]	0.649
	INVOS < 75	14.5 [14–15]		14.3 [13.6–154]		14.9 [13.8–15.3]		14.1 [13.6–14.8]	
NME (IU)	INVOS ≥ 75	26.5 [24–30]	0.234	21 [17.2–27.6]	0.886	19.3 [13.8–26.7]	0.919	17.6 [12.7–26.9]	0.956
	INVOS < 75	28.1 [26–30]		20.3 [17.6–27.8]		20.9 [14.8–26.5]		16.7 [13.5–25.5]	
HPDJ (IU)	INVOS ≥ 75	0.4 [0.37–0.42]	0.528	0.35 [0.32–0.4]	0.956	0.41 [0.35–0.51]	0.403	0.44 [0.33–0.52]	0.551
	INVOS < 75	0.41 [0.39–0.42]		0.36 [0.31–0.38]		0.45 [0.36–0.52]		0.49 [0.38–0.5]	
S100 (IU)	INVOS ≥ 75	219 [208–233]	0.301	264 [231–269]	0.321	112 [84.7–232]	0.946	627 [36.1–1710]	0.730
	INVOS < 75	226 [210–236]		254 [236–273]		119 [83.9–230]		40 [37–174]	
APOE (IU)	INVOS ≥ 75	690 [570–760]	0.715	920 [850–952]	0.976	910 [740–945]	0.150	815 [710–869]	0.643
	INVOS < 75	701 [550–765]		920 [810–948]		920 [840–984]		831 [730–889]	
TNF-α (IU)	INVOS ≥ 75	7.55 [7.34–7.92]	0.594	9.35 [9.09–9.66]	0.896	9.23 [8.87–9.55]	0.452	8.01 [7.42–8.53]	0.344
	INVOS < 75	7.58 [7.24–7.78]		9.37 [9.12–9.57]		9.27 [92–9.84]		8.21 [7.61–8.68]	
IL1 (IU)	INVOS ≥ 75	24.3 [23–26]	0.494	28.7 [27.6–30]	0.739	34.5 [32.5–36.5]	0.970	38.2 [35.3–39.7]	0.178
	INVOS < 75	24.1 [23–25]		28.6 [27.2–30.3]		34.7 [32.5–36.6]		36.9 [35.6–38.8]	
MMP9 (IU)	INVOS ≥ 75	472 [451–500]	0.651	523 [479–682]	0.739	728 [703–780]	0.673	717 [691–760]	0.620
	INVOS < 75	470 [433–490]		552 [477–660]		735 [700–748]		719 [683–753]	
IL6 (IU)	INVOS ≥ 75	282 [265–302]	0.611	296 [287–307]	0.391	301 [294–316]	0.007	305 [278–336]	0.044
	INVOS < 75	290 [266–304]		299 [283–313]		316 [298–334]		321 [309–347]	
IL8 (IU)	INVOS ≥ 75	97 [92–107]	0.736	198 [183–217]	0.891	122 [108–129]	0.869	248 [197–343]	0.584
	INVOS < 75	98 [92–106]		198 [183–216]		122 [107–129]		288 [193–383]	
IL10 (IU)	INVOS ≥ 75	0.1 [0.08–0.1]	0.459	0.1 [9–0.1]	0.175	0.1 [9–0.11]	0.891	0.1 [9–0.1]	0.627
	INVOS < 75	0.1 [0.09–0.1]		0.1 [9–0.11]		0.1 [9–0.11]		0.1 [9–0.11]	
MCP1 (IU)	INVOS ≥ 75	245 [241–250]	0.572	257 [252–263]	0.878	339 [328–343]	0.599	351 [344–356]	0.135
	INVOS < 75	243 [240–246]		258 [251–263]		340 [327–342]		352 [350–357]	
MMP2 (IU)	INVOS ≥ 75	231 [227–238]	0.767	327 [320–339]	0.77	365 [346–386]	0.495	427 [411–442]	0.66
	INVOS < 75	231 [223–237]		321 [310–331]		362 [349–380]		430 [415–453]	
MMP3 (IU)	INVOS ≥ 75	15.3 [14.5–17]	0.230	24.9 [22.8–26.7]	0.904	27.3 [25.5–28.4]	0.508	28.5 [26.9–29.1]	0.660
	INVOS < 75	15.2 [13–16]		25.1 [22.4–26.8]		26.7 [24.8–28.4]		28.6 [26.8–29.1]	
IL18 (IU)	INVOS ≥ 75	1.66 [1.5–1.8]	0.896	1.85 [1.75–1.96]	0.227	1.98 [1.94–2.5]	0.810	2.97 [2.84–34]	0.38
	INVOS < 75	1.7 [1.5–1.8]		1.79 [1.63–1.93]		1.97 [1.93–2.4]		2.88 [2.76–2.96]	

Abbreviations: INVOS = Intraoperative cerebral oximetry index; OLV = One-lung ventilation; PO = Postoperative; NSE = Neuron-specific enolase; NME = Neuron-specific microtubule-associated protein; HPDJ = Heat shock protein DJ-1; S100 = S100 calcium-binding protein; APOE = Apolipoprotein E receptor; TNF = Tumor necrosis factor; IL = Interleukin; MMP = Matrix metalloproteinase; MCP1 = Monocyte chemoattractant protein-1.

**Table 4:** Postoperative outcomes according to INVOS values.

Variable	INVOS ≥ 75 (*n* = 38) (*n* = 38)	INVOS < 75 (*n* = 60) (*n* = 60)	*p*-Value
Length of hospital stay (days)	4 [3–6]	5 [4–7]	0.019
Baseline mini-mental score	30 [29–30]	29 [28–30]	0.23
Mini-mental score at 72 h	30 [29–30]	29 [27–30]	0.018
Patients with mini-mental score at 72 h ≤ 26 points	1 (2.7%)	9 (15.3%)	0.047
Patients with postoperative mini-mental drop ≥ 2 points	2 (5.4%)	13 (22%)	0.025
Percentage change in mental score	0 [0–0]	0 [−0.3–0]	0.046
Clavien–Dindo classification			0.112
No complications	27 (71.1%)	33 (55%)	
Minor complications	11 (28.9%)	23 (38.4%)	
Severe complications	0 (0%)	4 (6.6%)	
Postoperative cardiac complication	0 (0%)	2 (3.3%)	0.372
Renal insufficiency	0 (0%)	2 (3.3%)	0.372
Any postoperative pulmonary complications.	8 (21.1%)	18 (30%)	0.328
Respiratory failure	1 (2.6)	5 (8.3%)	0.245
Atelectasis	4 (10.5%)	7 (11.7)	0.568
Bronchoscopic intervention	0 (0%)	2 (3.3%)	0.372
Pneumonia	0 (0%)	4 (6.6%)	0.135
Non-invasive mechanical ventilation	0 (0%)	2 (3.3%)	0.372
Respiratory infection	6 (15.8%)	9 (15%)	0.916
Pulmonary embolism	0 (0%)	1 (1.7%)	0.805
Atrial fibrillation	3 (7.9%)	7 (11.7)	0.407
Pleural effusion	2 (5.3%)	11 (18.3%)	0.056
Any surgical complications	3 (7.9%)	10 (16.5%)	0.174
Wound infection	0 (0%)	3 (5%)	0.225
Hospital readmission	2 (5.3%)	5 (8.3%)	0.443
Mortality at 1 year	0 (0%)	2 (3.3)	0.372
Mortality at 2 years	1 (2.6%)	5 (8.3%)	0.245

## Data Availability

Data available on request due to restrictions (regarding patient confidentiality).

## Abbreviations

The following abbreviations are used in this manuscript:

CPAP	Continuous positive airway pressure
FiO_2_	Inspired fraction oxygen
H-INVOS	High-INVOS
IL	Interleuquine
INVOS	In Vivo Optical Spectroscopy
L-INVOS	Low-INVOS
MMPs	Metalloproteinases
MMSE	Mini-Mental State Examination
OLV	One-lung ventilation
PEEP	Positive end-expiratory pressure
POCD	Postoperative cognitive dysfunction
rScO_2_	Regional cerebral oxygen saturation
SpO_2_	Peripherical saturation oxygen
TNF	Tumor necrosis factor
TV	Tidal volume
